# Clozapine-associated eosinophilia with multiple systemic involvement - case report and review of literature

**DOI:** 10.1192/j.eurpsy.2021.1289

**Published:** 2021-08-13

**Authors:** I. Orlović, T. Sugnet, M. Peco, V. Golubić Zatezalo, H. Karlica, D. Karlović

**Affiliations:** 1 Department Of Psychiatry, Sestre Milosrdnice University Hospital Center, Zagreb, Croatia; 2 Department Of Psychiatry, University Hospital Center Split, Split, Croatia

**Keywords:** clozapine, eosinophilia, multiorgan dysfunction

## Abstract

**Introduction:**

Due to its mood-stabilizing properties, clozapine is known for reducing symptom severity in manic episodes of treatment-resistant bipolar disorder as well as in treatment-resistant schizophrenia. However, its use may be hindered by potential adverse effects, including hematologic ones, such as non-dose-dependent eosinophilia. The mechanism of the underlying process probably involves a type-I hypersensitivity reaction, which can manifest as either transient asymptomatic eosinophilia or as eosinophilia with multiorgan dysfunction.

**Objectives:**

We present the case of a patient diagnosed with manic episode of schizoaffective disorder who developed eosinophilia, with severe systemic manifestations, in response to clozapine therapy. A review of literature will be conducted in order to provide further insight into the phenomenon.

**Methods:**

Case report and literature review.

**Results:**

The incidence of eosinophilia reported in literature ranges between 0.2% and 62%, with its appearance about three weeks after clozapine initiation. Although clozapine is an antipsychotic that normally requires frequent monitoring due to the potential side effect of agranulocytosis, we would like to place emphasis on the possible risk of eosinophilia, in connection with potential fatal complications. As described in this report, eosinophilia could long remain unrecognized due to subsequent multiorgan involvement, including lymphadenopathy, leukocytosis, lymphopenia, anemia, liver enzyme elevations, as well as pleural effusion, all of which were described in our patient.

**Conclusions:**

Clozapine-associated eosinophilia may be used as an early marker of possible clozapine-induced systemic complications and it may warrant prompt discontinuation of the causing drug, as suggested by the literature.

